# Valve morphology and timing of surgery in bicuspid aortic valve disease

**DOI:** 10.1007/s00392-025-02725-1

**Published:** 2025-08-19

**Authors:** Sandra Wulffeld, Michelle Dalgas Skøtt Schmiegelow, Riina Oksjoki, Dorte Guldbrand Nielsen, Søren Skøtt Schmiegelow, Anh Thuc Ngo, Jakob Raunsø, Morten Kranker Larsen, Niels Eske Bruun, Kristina Procida

**Affiliations:** 1 Department of Cardiology, Zealand University Hospital, Roskilde, Denmark; 2 Department of Cardiology, Rigshospitalet, Copenhagen University Hospital, Copenhagen, Denmark; 3 Department of Cardiology, Aarhus University Hospital, Aarhus, Denmark; 4 Department of Cardiology, Herlev-Gentofte Hospital, Herlev, Denmark; 5 Department of Hematology, Zealand University Hospital, Roskilde, Denmark; 6 Faculty of Health and Science, Department of Clinical Medicine, Copenhagen University, Copenhagen, Denmark; 7 Institute of Clinical Medicine, Aalborg University, Aalborg, Denmark

**Keywords:** Bicuspid aortic valve, Heart valve disease, Aortic dilatation, Aortic valve surgery, Aortic surgery

## Abstract

**Background:**

The anatomy of bicuspid aortic valves (BAV) varies considerably and is broadly classified into two main types: two-sinus and fused BAV. Possible prognostic implications of these two main types remain unclear. This study aimed to assess potential associations between BAV morphology and the timing of surgery of the aortic valve or ascending aorta.

**Methods:**

A multi-center cohort study including 1004 adult outpatients with BAV. BAV morphology was classified as either two-sinus or fused type. The primary outcome was a composite of surgical or endovascular intervention on the aortic valve or ascending aorta. The effect of morphology on the primary outcome was investigated using delayed-entry cause-specific Cox regression models using age as timescale.

**Results:**

A total of 835 patients with fused BAV and 169 with two-sinus BAV were followed for 2044 person-years. Two-sinus BAV patients were younger (median age 47.2 vs. 53.6 years, *p* = 0.0002) with a higher prevalence of aortic coarctation (24% vs. 12%, *p* = 0.0003). The incidence rate of surgery was 9.3 per 100 person-years in fused BAV patients and 10.7 per 100 person-years in two-sinus BAV patients (difference [95%CI]: 1.42 [− 2.3, 5.1] per 100 person-years). Two-sinus morphology was associated with a higher age-related hazard of surgery compared to fused BAV (HR [95%CI]: 1.46 [1.02, 2.09]), a finding that remained significant after adjusting for sex.

**Conclusion:**

Two-sinus BAV morphology was associated with a significantly higher age-related likelihood of requiring surgery on the aortic valve or ascending aorta.

**Graphical Abstract:**

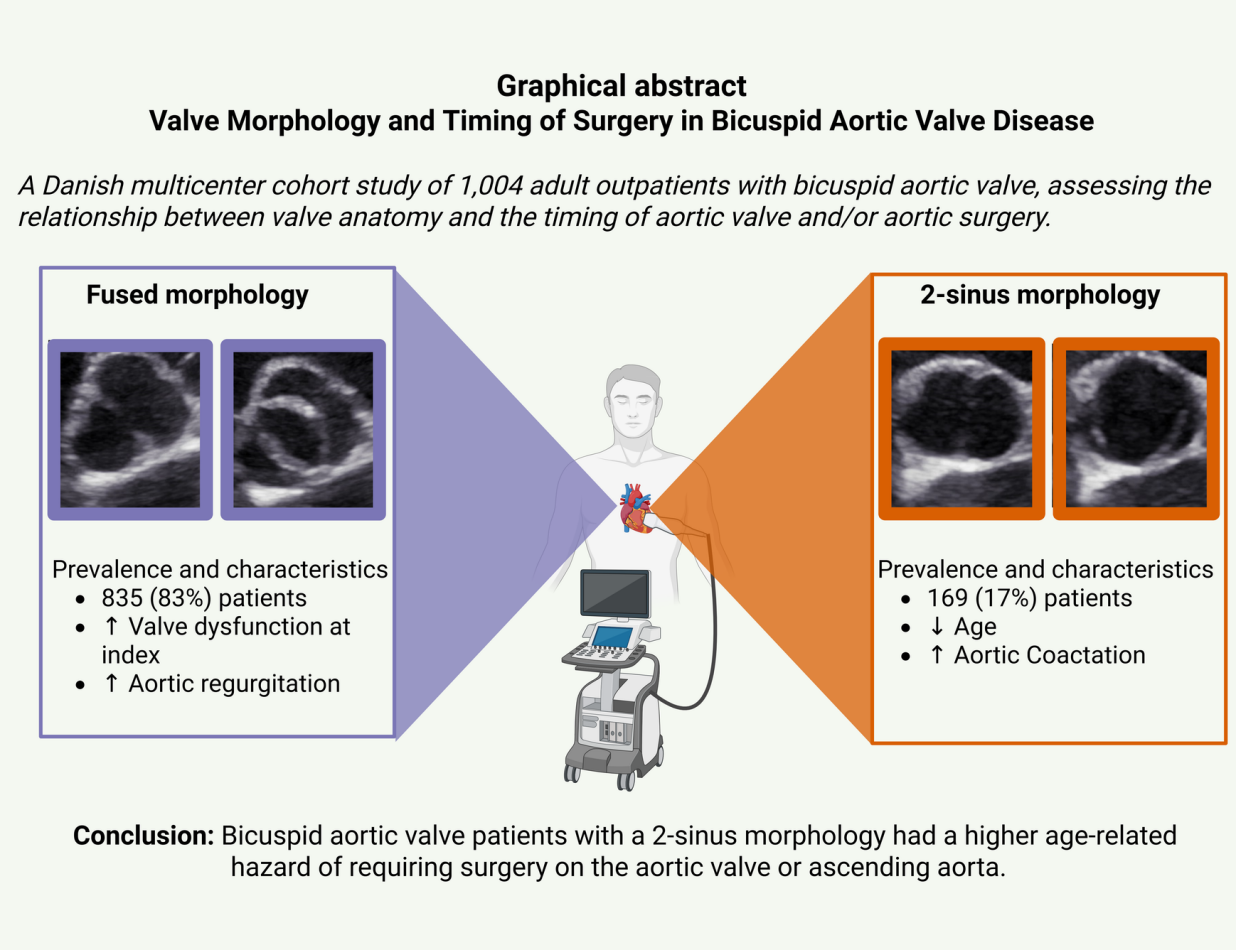

**Supplementary Information:**

The online version contains supplementary material available at 10.1007/s00392-025-02725-1.

## Introduction

Approximately 1% of the Danish population is born with a bicuspid aortic valve (BAV), which is a risk factor for early-onset aortic valve dysfunction and dilatation of the ascending aorta [1–3]. If these conditions become severe, patients need surgical or endovascular intervention to prevent life-threatening complications such as heart failure, sudden cardiac death, or aortic dissection or rupture. Among outpatients with BAV, the estimated cumulative incidence of surgery of the aortic valve or aorta is 69.2% by 90 years of age [4]. However, a substantial proportion of people with BAV remain undiagnosed and, presumably, never develop symptomatic complications. This variability in disease progression presents a clinical challenge, particularly when BAV is an incidental finding in patients without significant valve disease or aortic dilatation at diagnosis. To address this challenge, we need evidence supporting individual risk estimation of BAV patients to enable rational planning of a clinical surveillance program. Hence, we must expand our understanding of patient-specific factors related to the development of severe complications in BAV disease.

Factors with potential prognostic significance in BAV patients are the specific morphological features of the malformed valve. Although evidence is sparse, some observations offer plausible hypotheses that could relate anatomy to the clinical course of BAV disease. First, animal studies have related BAV morphology to disturbance in specific stages during aortic valvulogenesis [5, 6]. A link between the severity of the embryologic defect and morphology could explain why patients with comparable BAV anatomy would share a similar prognosis. Secondly, a pathophysiological mechanism of aortic dilatation is suggested by the observation from flow-MRI studies, that BAV valve anatomy is associated with flow disturbances in the ascending aorta [7]. Furthermore, a fibrous commissural ridge, termed a raphe, may serve as an area prone to early calcification [8, 9].

Whether morphological features relate to prognosis in BAV disease has been extensively investigated but remains unresolved. Previous studies have mainly focused on the possible association between BAV anatomy and echocardiographic variables. Findings from these studies are inconsistent [10, 11]. Prognostic studies using clinical endpoints are sparse [8, 12]. Additionally, confusing nomenclature of BAV anatomy may have contributed to a lack of scientific progress. To address these issues, international experts recently established a uniform classification system for BAV anatomy (Consensus Classification) [13]. In this study, our aim is to examine whether BAV morphology, as defined by the Consensus Classification, is associated with the age-adjusted likelihood of requiring surgery on the aortic valve or ascending aorta.

## Methods

### Study population

In this multicenter cohort study, we identified all adults with BAV from the outpatient clinics of three major departments of cardiology in Denmark during three department-specific accrual periods: 2018–2020 (Zealand University Hospital), 2019–2020 (Aarhus University Hospital (AUH)), and 2020–2022 (Herlev-Gentofte Hospital). Participating departments have dedicated outpatient clinics for patients with simple congenital heart disease, providing both pre-surgical clinical surveillance and post-surgical follow-up. Furthermore, AUH is a tertiary hospital with highly specialized cardiology functions, including heart surgery and a complex congenital heart disease clinic for both pediatric and adult patients. To identify possible BAV cases, we screened electronic health charts of patients with active affiliation to outpatient clinics for congenital heart disease and patients with an ICD-10 code of BAV or congenital aortic valve disease. Next, we reviewed echocardiograms in our imaging databases to visually confirm the BAV diagnosis. We defined BAV by the presence of a fish mouth–shaped orifice or only two distinct cusps.

To identify a prospective cohort from this mixed population of BAV outpatients, we included all patients with an available echocardiogram performed after 31^st^ December 2017, since this was the beginning of the accrual period. Exclusion criteria were prior surgery of the aortic valve or ascending aorta, genetic syndromes, concomitant congenital heart malformations except unrepaired ventricular or atrial septal defects, and age < 18 years. Prior surgical correction of aortic coarctation was not an exclusion criterion. We also excluded patients with a unicuspid valve and patients with an indeterminate anatomical classification. Lastly, we excluded 12 patients with partial fusion BAV.

### Outcomes

The primary outcome was a composite endpoint including any interventional procedure—both endovascular and surgical—targeting the aortic valve or ascending aorta. This included aortic valve replacement or repair, and aortic reconstruction, but excluded procedures exclusively correcting aortic coarctation. Procedures with concomitant coronary artery bypass grafting or percutaneous coronary intervention were included. This primary outcome will be referred to as surgery of the aortic valve or ascending aorta. In each patient, we registered the time from the date of the first available echocardiogram in the study period (index TTE) until the date of surgery of the aortic valve or ascending aorta, death, or end of observation, whichever came first. The last observation for each patient was the date of the most recent review of their electronic health chart. For the whole study population, the last observation was during the period March–December 2022.

### Data sources

We retrieved data by reviewing electronic health journals and imaging databases (EchoPAC and Viewpoint (GE Healthcare), Impax (AGFA)) and collected data in a secure RED-Cap database hosted by Region Zealand [14, 15]. Since the electronic health journals in Denmark cover all hospital contacts within the same region and receive discharge letters from all other hospitals, we expect a highly sensitive detection of clinical events. Furthermore, the electronic health journal is linked to the Danish Civil Registration System and automatically registers all deaths.

### BAV morphology

BAV morphology was categorized both according to major BAV type (fused vs. two-sinus BAV) and by specific phenotypes as defined in the Consensus Classification [13]. For a comprehensive introduction to the Consensus Classification and how it relates to the universally used classification as suggested by Sievers and Schmidtke, we refer to the original statement [13, 16]. In brief, the major BAV types differ by the shape of the aortic root and the symmetry and angulation of the two cusps. A fused BAV has a three-sinus-shaped aortic root, asymmetric cusps, and possibly a visible raphe. The aortic root of the two-sinus BAV is nearly oval with only two distinct sinuses, and the cusps are more symmetric. Compared to the Sievers and Schmidtke classification, the major changes in the Consensus Classification are that Sievers type 2 with two raphes is no longer considered a bicuspid valve. Furthermore, a visible raphe, which is a prerequisite for Sievers type 1 classification, is not essential for the morphology to be considered a fused BAV. As a result, all two-sinus BAVs are expected to be classified as Sievers type 0, while the fused BAV group likely includes both patients previously classified as Sievers type 0 and type 1. The major BAV types of the Consensus Classification may be further subdivided into specific phenotypes based on the spatial orientation of the commissures (see Figure 2). To ensure correct anatomical classification, we reviewed all available transthoracic and transesophageal echocardiograms of each patient. We further noted the visibility of the raphe.

### Echocardiographic variables

All echocardiograms were routinely performed during clinical check-ups in the out-patient clinics. For the purpose of this study, examinations were re-reviewed off-line. Assessors were primarily consultant cardiologists working in one of the BAV outpatient clinics or one cardiology resident with approximately 2 years of echocardiography training and practical experience. In cases of uncertainty, consensus was reached between at least two assessors. To assess differences between the anatomical subgroups at baseline, we report the presence and grade of valve dysfunction, aortic dilatation, and left ventricular ejection fraction (LVEF) at index TTE. The grade of aortic stenosis (AS) was assessed using the mean/max pressure gradients across the valve and the aortic valve area (AVA) as calculated by the continuity equation. If calculation by the continuity equation was not accessible, we estimated AVA by planimetry, if a high-quality transesophageal echocardiogram was available. Since pressure gradients and AVA could not always be measured at the same echocardiogram, we categorized AS severity as either moderate-to-severe stenosis or absent-to-mild stenosis. We defined moderate-severe stenosis as the presence of at least one of the following: mean pressure gradient ≥ 20 mmHg, max pressure gradient ≥ 40 mmHg, or AVA < 1.5 cm^2^. Likewise, no more than mild AS was confirmed if at least one of the variables (pressure gradients or AVA) was available, but measurements were below the threshold for moderate-severe AS. If both pressure gradients and AVA measures were unavailable, the AS variable was registered as missing. The presence and grade of aortic regurgitation (AR) were re-assessed semi-quantitatively in accordance with current guidelines [17]. Left ventricular ejection fraction (LVEF) was estimated using the Simpsons biplane method and categorized as follows: normal (LVEF > 55%), mildly reduced (LVEF 40-55%), moderate-severely reduced (LVEF < 40%). The ascending aorta was measured in the parasternal long-axis view at end-diastole from leading-edge to leading-edge. We measured the cross-sectional diameter at sinus of Valsalva, sinotubular junction, and the broadest part of the visible tubular ascending aorta. We defined aortic dilatation by a measurement of at least 39 mm at any segment.

### Clinical variables

Finally, we registered date of birth, sex, aortic coarctation, and concomitant heart defects. We noted whether patients reported a family history of confirmed or suspected BAV, sudden cardiac death, or aortic dissection. From the procedural reports, we noted the primary indication for the procedure on the aortic valve or proximal aorta and the type of intervention.

### Statistics

After assessing their distribution, continuous variables are summarized using median [interquartile range] or mean [SD] as appropriate and compared using Wilcoxon-rank-sum test or *t* test as appropriate. Categorical or binary variables are presented as counts [percentage] and compared using chi-square tests. The frequency of the primary outcome is described using the incidence rate per 100 person years overall and according to major BAV type.

To assess the association between BAV anatomy and the primary outcome, we estimated the hazard ratio with 95% confidence intervals (HR [95%CI]) in both simple and sex-adjusted cause-specific Cox regression models using age as timescale. Since study inclusion was dependent on patients surviving until their age at index TTE, we accounted for this delayed entry in the Cox models. Consequently, each patient only contributed observation time to the models starting from their age at the index TTE. Observation continued until either the age at time of surgery or the age at censoring (death or end of observation). In all analyses, we used the largest subgroup, major BAV type (fused), or phenotype (fused R-L phenotype), as the reference for comparison. The null hypothesis of no difference in hazards between subgroups was assessed by Wald’s test. The proportional hazards assumption (i.e., that the hazard ratio remains constant across all ages in our study) was assessed by testing the independence between time and Schönfeld residuals for each variable, as well as for the global multivariate model. Furthermore, estimated cumulative hazard was visualized using Nelson-Aalen curves. To evaluate whether the association between major BAV type and timing of surgery differed by sex, we tested for the presence of interaction between BAV type and sex using the likelihood ratio test.

We conducted several secondary analyses to investigate potential factors that may mediate the association between BAV morphology and timing of surgery. First, we hypothesized that any observed association would be mediated by development of valve dysfunction or aortic dilatation at an earlier age. Therefore, we visualized the prevalence of moderate-to-severe valve dysfunction and aortic dilation > 39 mm at index TTE across three age strata (18–40 years, 40–60 years, and > 60 years) and by BAV morphology. Associations between BAV type and moderate-severe valve dysfunction or aortic dilatation were assessed in three logistic regression analyses adjusting for age. Secondly, we conducted several univariate Cox regression analyses to evaluate potential associations between the age-specific hazard of surgery and the following risk factors: male sex, aortic coarctation, and the following findings on index echocardiogram: moderate-severe valve dysfunction, aortic dilatation > 39 mm at any segment, and visible raphe. In all hypothesis tests, we considered a two-sided *p* value < 0.05 as significant. All data processing, statistical analyses, and visualization were performed using R version 4.4.2 [18].

## Results

### Clinical and echocardiographic characteristics

**Fig. 1:**
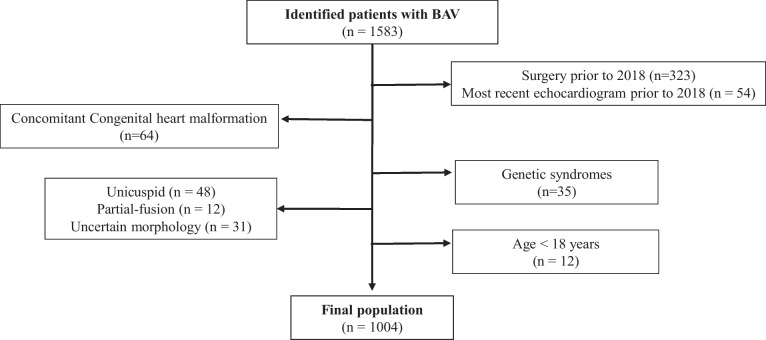
Flow chart of study inclusion.* BAV* bicuspid aortic valve

We identified 1583 patients with BAV, and following application of the exclusion criteria, the final study population comprised 1004 adult BAV outpatients with a median age of 52.2 years and 73.2% males (Figure 1; Table 1). In total, the population was observed for 2137 person-years (1782 person-years in patients with fused BAV, 356 person-years in patients with two-sinus BAV) corresponding to a median follow-up time of 2.2 years, which did not differ between morphological subgroups.

**Table 1:** Morphological features and clinical characteristics according to major BAV type

Variables	Total (*n* = 1004)	Fused (*n* = 835)	2-sinus (*n* = 169)	*p*
Raphe	-	637 (76.4)	-	
Age	52.2 [38.8, 63.7]	53.6 [40.3, 64.0]	47.2 [31.2, 60.0]	0.0002
Male sex	735 (73.2)	612 (73.3)	123 (72.8)	1
Coarctation of the aorta	139 (14.0)	99 (12.0)	40 (24.0)	0.0003
Previous CoA repair	123 (88.5)	84 (84.8)	39 (97.5)	0.04
Minor septal defects				0.07
Atrial septal defect	5 (0.5)	3 (0.4)	2 (1.2)	
Ventricular septal defect	17 (1.7)	11 (1.3)	6 (3.6)	
BAV family history	110 (11)	95 (11.4)	15 (8.9)	0.4
SCD/aortic dissection family history	54 (5.4)	42 (5)	12 (7.1)	0.5
Age at diagnosis	46.3 [26.3, 58.2]	47.4 [28.7, 58.8]	41.9 [14.4, 53.1]	0.0003

Anatomical BAV characteristics and basic clinical data of the study population. Numbers are count (percentage) or median [IQR]. *P* values are from the chi-square test or Wilcoxon-rank-sum test

*SCD* sudden cardiac death, *CoA* coarctation of the aorta

**Fig. 2:**
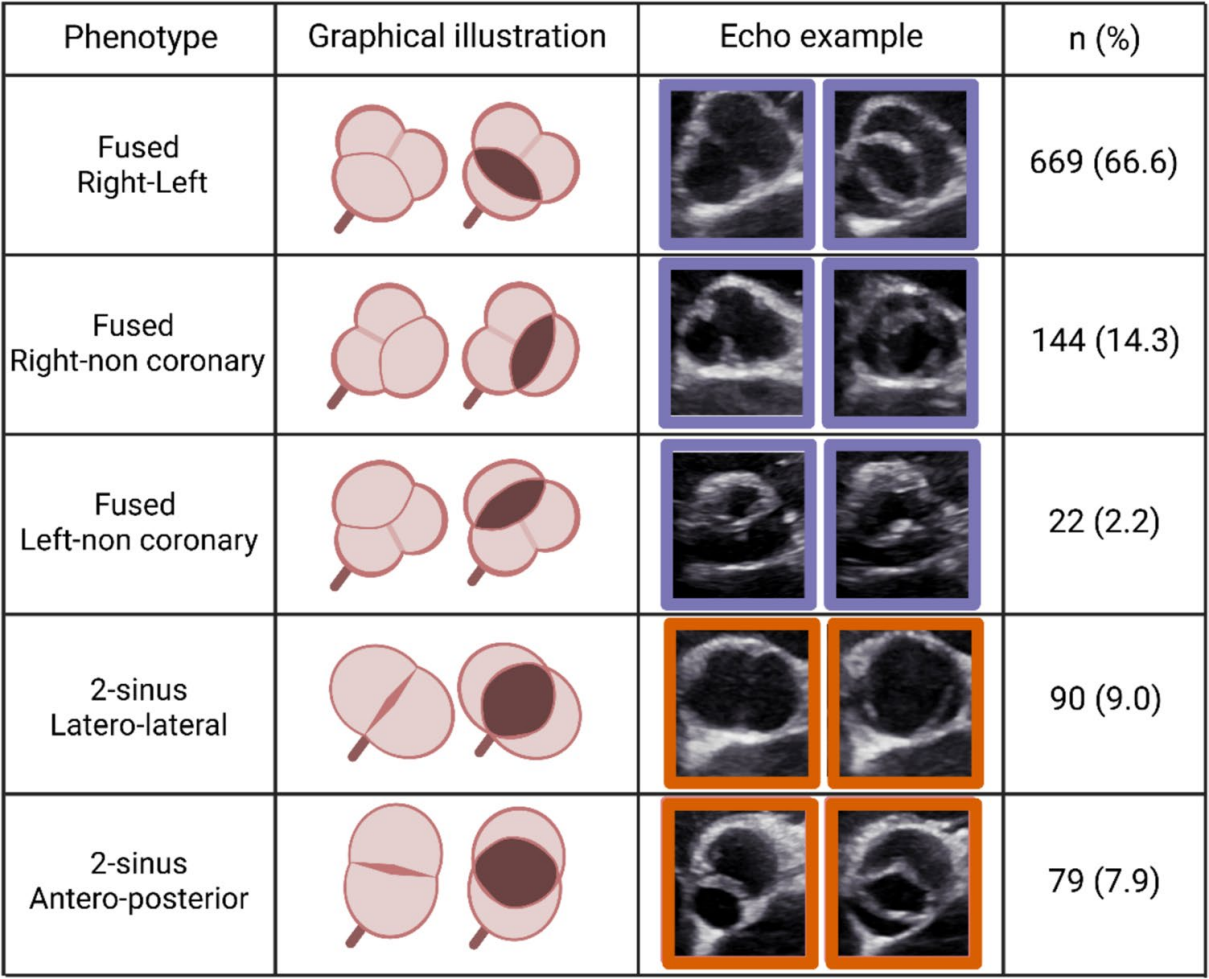
Morphology and distribution of specific valve phenotypes. Images are from transthoracic echocardiography, short-axis view

Most patients in the study population had a fused BAV (83.2%) of whom a raphe was visible in 76.4%. Considering the specific phenotypes, two-thirds of the patients (66.6%) had a fused R-L phenotype, whereas the fused L-N phenotype was found in only 2.2% of the total study population (Figure 2). Patients with a 2-sinus BAV were significantly younger at index compared to the fused BAV subgroup (Table 1). Concomitant aortic coarctation was twice as frequent in patients with the two-sinus BAV as in the fused BAV subgroup, and particularly prevalent in patients with the two-sinus anteroposterior phenotype, with an observed proportion of 43% (supplementary table S1). Minor septal defects were rare, occurring in only 22 (3.2%) patients, but appeared to be more frequent in the two-sinus subgroup (*p* = 0.07). The family medical history was accessible in 77% of patients and 11% reported a possible family history of BAV, whereas 5.4% reported a family history of known or suspected cases of sudden cardiac death or aortic dissection. There were no significant differences in reported family medical history between the anatomical subgroups (Table 1).

On the index echocardiogram, 45.5% had minimal or no aortic valve dysfunction, primarily in the two-sinus subgroup (52.1% vs. 44.2%, *p* = 0.02). Moderate–severe aortic regurgitation was more common in the fused BAV subgroup (21% vs. 9.6%, *p* = 0.003). However, there were no significant differences in the occurrence of aortic stenosis, LVEF, or aortic dilatation (Table 2). Detailed information on echocardiographic variables and characteristics according to the specific phenotypes are available as supplementary material (Supplementary Tables 1 and 2).

**Table 2:** Findings on index echocardiogram according to major BAV type. Aortic valve function, left ventricular systolic function, and presence of aortic dilatation at any proximal segment on index echocardiogram. Numbers are count (percentage). p values are from the chi-square test. LVEF left ventricular ejection fraction

Variables	Total	Fused BAV	2-sinus BAV	*p*
Moderate-severe aortic valve dysfunction				
None	457 (45.5)	369 (44.2)	88 (52.1)	
Aortic regurgitation	113 (11.3)	102 (12.2)	11 (6.5)	
Aortic stenosis	321 (32.0)	265 (31.7)	56 (33.1)	
Aortic regurgitation and stenosis	72 (7.2)	67 (8.0)	5 (3.0)	0.02
LVEF				
Normal	860 (85.8)	725 (86.9)	135 (80.4)	
Mildly reduced (40-55%)	106 (10.6)	78 (9.4)	28 (16.7)	
Moderate-severely reduced (<40%)	32 (3.2)	28 (3.4)	4 (2.4)	0.08
Aortic dilatation > 39 mm	589 (62.2)	491 (62.5)	98 (60.5)	0.7

### Association between BAV morphology and surgery of the aortic valve or ascending aorta

By the end of observation, 20.2% (*n* = 203) of all patients had undergone surgery of the aortic valve or ascending aorta. The incidence rate of this primary event was 9.3 per 100 person-years in fused BAV patients and 10.7 per 100 person-years in two-sinus BAV patients (estimated rate difference [95%CI]: 1.42 [− 2.3, 5.1] per 100 person-years). During follow-up, 25 patients died: one patient with a two-sinus BAV (0.3 per 100 person years) and 24 with a fused BAV (1.4 per 100 person-years). Median age at time of death was 65.4 years IQR: [58.8, 72.5].

**Fig. 3:**
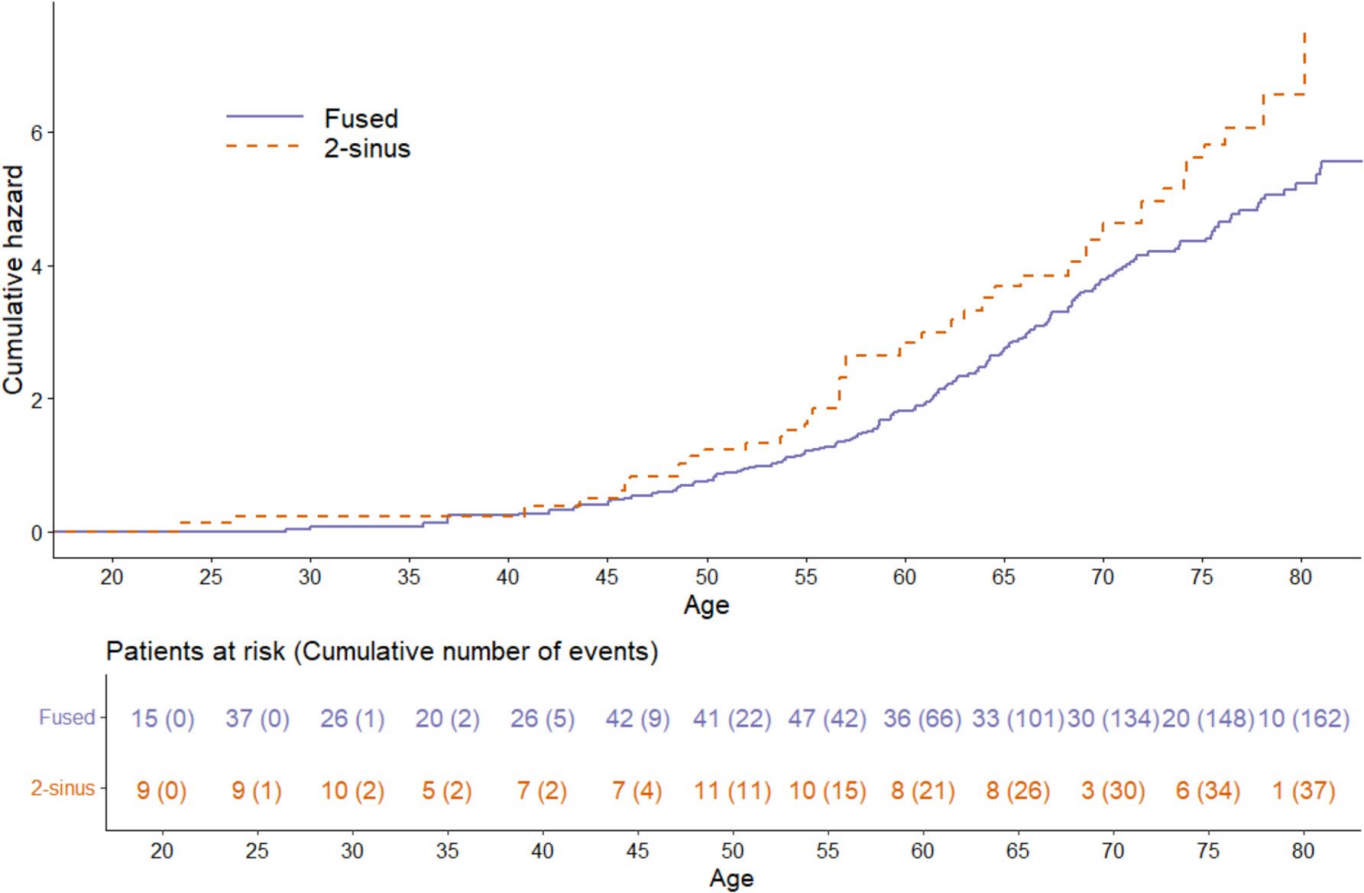
Nelson-Aalen cumulative hazard of aortic valve or aortic surgery by bicuspid aortic valve (BAV) morphology. The solid blue line represents patients with fused BAV, while the dashed red line represents those with two-sinus BAV. The table displays the number of patients at risk at increasing ages, with risk sets varying due to delayed entry. Numbers in parenthesis are the cumulative number of events

**Fig. 4:**
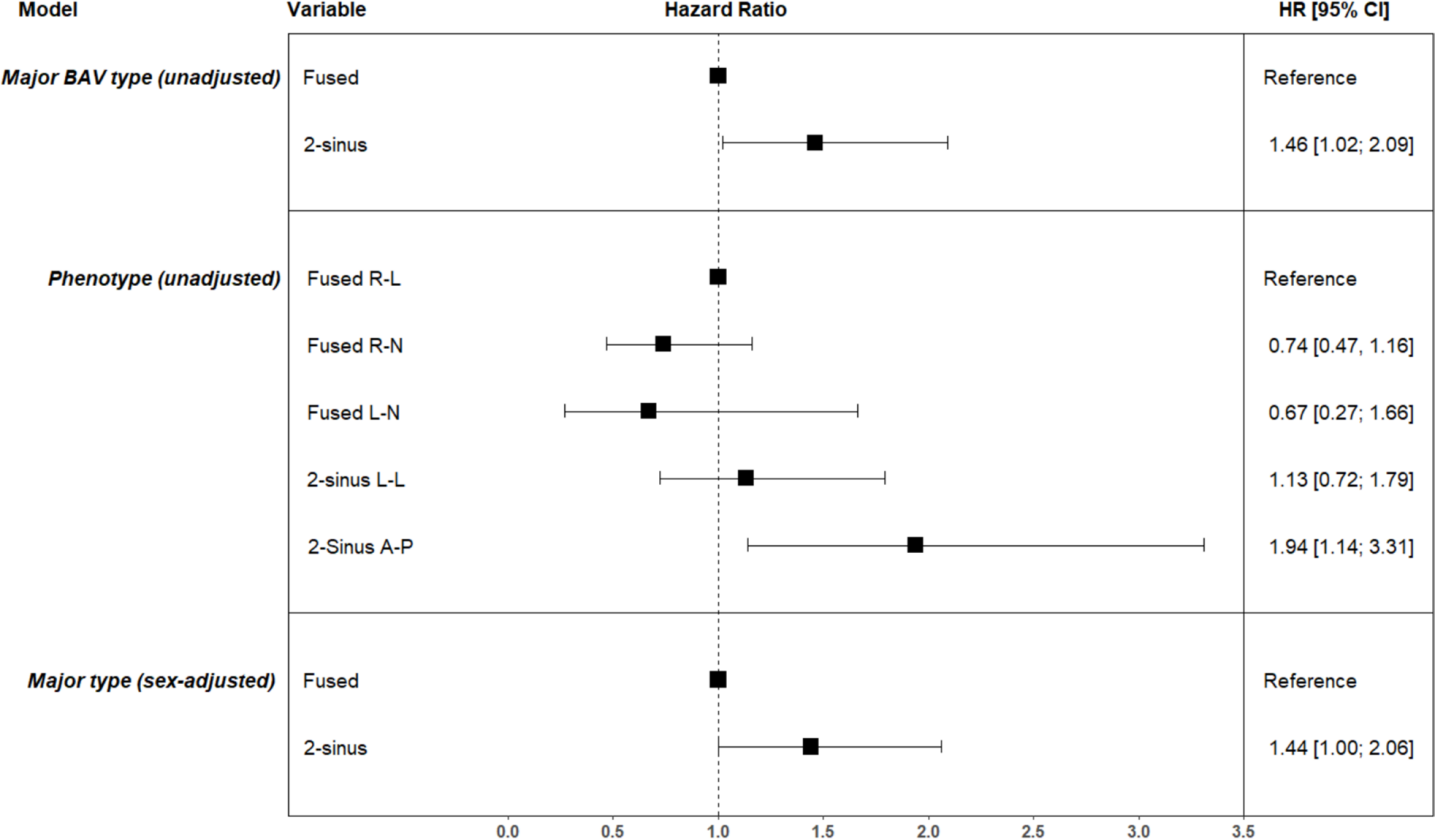
Primary analyses. Univariate (two upper panels) and sex-adjusted (lower panel) cause-specific hazard of surgery of the aortic valve or ascending aorta. Delayed-entry cause-specific Cox regression models using age as timescale assessing the association between BAV anatomy and the hazard of surgery of the aortic valve or ascending aorta

In univariate analysis, two-sinus BAV morphology was associated with a higher hazard of surgery when compared to the fused BAV morphology (HR [95%CI]: 1.46 [1.02, 2.09]). The estimated cumulative hazard according to BAV type is shown in Figure 3. The curves overlap until approximately 45 years of age, after which they progressively diverge. This suggests that the hazard ratio is similar in younger patients, while the likelihood of requiring surgery increases more rapidly in patients with two-sinus BAV from mid-adulthood (Figure 3). The estimated HR did not change after adjustment for sex (HR [95%CI], 1.44 [1.00; 2.06]). When comparing the most common phenotype (fused R-L) to the other phenotypes, only the two-sinus A-P subgroup seemed associated with increased hazard of surgery (HR [95%CI], 1.94 [1.14, 3.31] (Figure 4).

Surgical aortic valve replacement was the most common intervention with only 18 cases of transcatheter aortic valve implantation (TAVI) and two cases of valve-sparing replacement of the ascending aorta. Aortic stenosis, with or without concomitant aortic dilatation, was the most frequent indication for intervention in both morphological subgroups. It accounted for 73.9% of procedures in fused BAV patients and as many as 84.2% among two-sinus patients (*p* = 0.2) (Table 3). Aortic regurgitation as an indication for surgery was more common in patients with fused-type (16.4% vs. 5.3% in two-sinus patients, *p* = 0.1). The association between surgery of the aortic valve or aorta and BAV morphology appeared stronger in women (HR (two-sinus versus fused BAV), 2.53 [1.26; 5.11] compared to 1.22 [0.80; 31.85] in men), but the interaction was not statistically significant (p for interaction = 0.09).

**Table 3:** Surgical details

Variables	Total (*n* = 203)	Fused BAV (*n* = 165)	2-sinus BAV (*n* = 38)	*p*
Median age at surgery	62 [54.0, 68.4]	62.4 [54.9, 68.4]	56.9 [49.3, 68.9]	0.2
Type of procedure				0.2
Bioprosthetic valve	77 (37.9)	65 (39.4)	12 (31.6)	
Bioprosthetic valve and aortic graft	22 (10.8)	17 (10.3)	5 (13.2)	
Mechanical valve	47 (23.2)	38 (23.0)	9 (23.7)	
Mechanical valve and aortic graft	20 (9.9)	15 (9.1)	5 (13.2)	
TAVI	18 (8.9)	14 (8.5)	4 (10.5)	
Composite graft	14 (6.9)	13 (7.9)	1 (2.6)	
Other	2 (1.0)	2 (1.2)	0 (0.0)	
Aortic graft only	2 (1.0)	0 (0.0)	2 (5.3)	
Primary indication for surgery				0.4
Aortic stenosis w/or w/o aortic dilatation	116 (75.9)	122 (73.9)	32 (84.2)	0.2
Aortic regurgitation w/or w/o aortic dilatation	29 (14.3)	27 (16.4)	2 (5.3)	0.1
Aortic dilatation	7 (3.4)	5 (3.0)	2 (5.3)	0.6
Mixed aortic stenosis and regurgitation	6 (3.0)	4 (2.4)	2 (5.3)	
Endocarditis	3 (1.5)	3 (1.8)	0 (0.0)	
Aortic dissection	2 (1.0)	2 (1.2)	0 (0.0)	

Procedural details of 203 patients receiving endovascular or surgical intervention of the aortic valve or proximal aorta during the follow-up period. Indication and procedure type as stated in the procedural report in the electronic health journal. Numbers are count (percentage) or median [IQR]

*TAVI* transcatheter aortic valve implantation

### Potential explanatory factors relating morphology to timing of surgery of the aortic valve or aorta

**Fig. 5:**
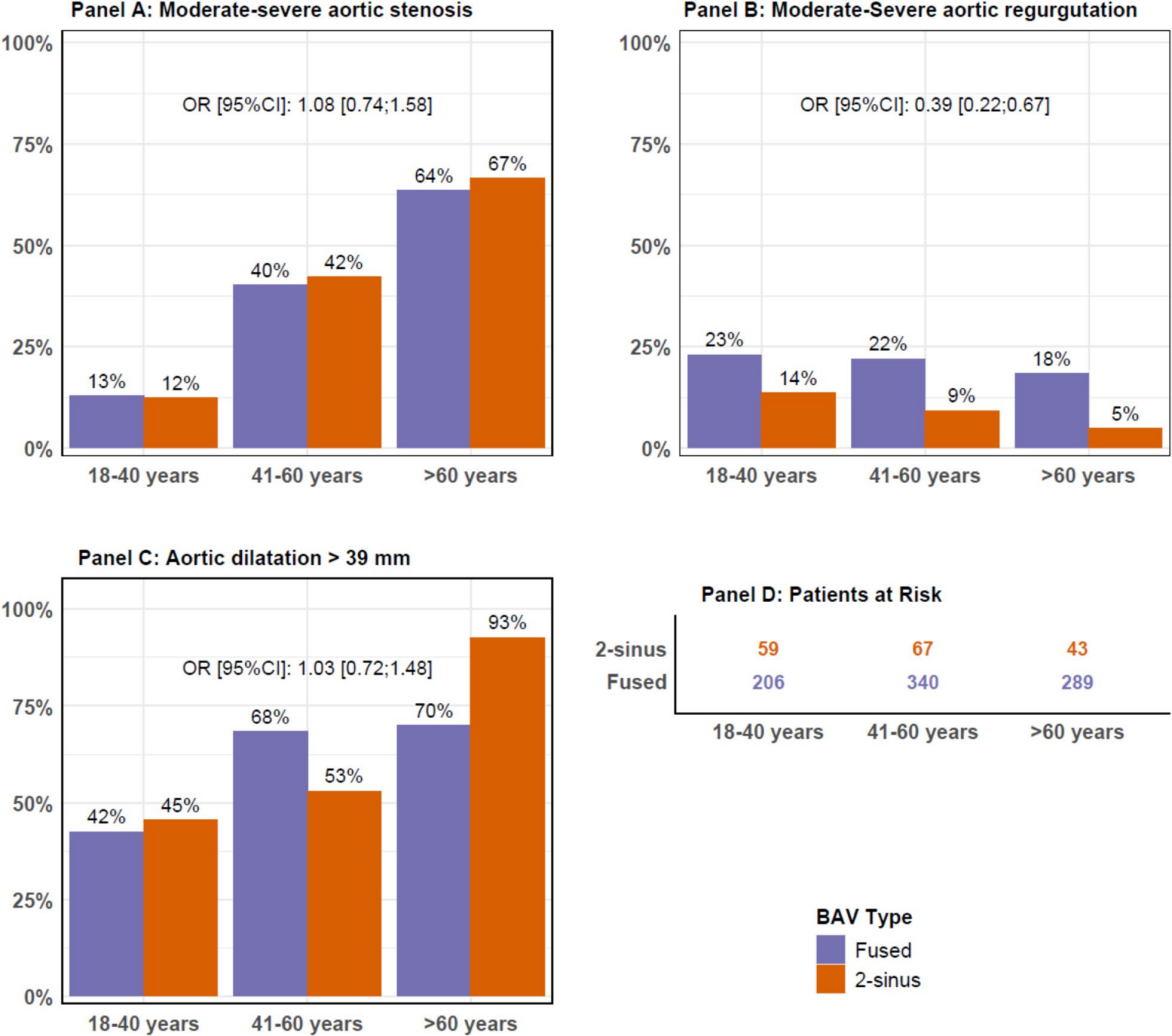
Observed frequency of moderate-severe aortic stenosis (**A**), moderate-severe aortic regurgitation (**B**), aortic dilatation > 39 mm at any segment (**C**) at index echocardiogram according to age-strata: 18–40 years, 41–60 years, and + 60 years. **D** Number of patients in each age-group. **A**–**C** Results from age-adjusted logistic regression analyses assessing the association between BAV type and each complication. OR, odds-ratio. CI, confidence interval

Figure 5 shows observed prevalence of BAV moderate-severe aortic valve dysfunction and aortic dilatation at index TTE according to age group and Major BAV type. In age-adjusted logistic regression analyses, we found no association between BAV type and moderate-severe aortic stenosis nor between BAV type and aortic dilatation > 39 cm, whereas fused BAV was associated with a significantly higher prevalence of moderate-severe aortic regurgitation in all age groups (age-adjusted OR (fused vs. two-sinus) [95%CI]: 2.64 [1.53, 4.55]).

Inunivariate Cox regression analyses, male sex and moderate-to-severe valve dysfunction at index TTE were both associated with the timing of surgery. The presence of a raphe, concomitant aortic coarctation, and aortic dilatation > 39 mm at index TTE were not significantly associated with the primary outcome (Table 4).

**Table 4:** Assessment of additional predictors and their association with the primary event

Explanatory variables	HR	95%CI	*p* value
Sex			
Female	Ref		
Male	1.60	[1.14; 2.26]	0.007
Moderate-severe valve dysfunction			
None	Ref		
Aortic regurgitation	15.25	[7.08;32.85]	<0.001
Aortic stenosis	22.56	[11.41;44.63]	<0.001
Aortic stenosis and regurgitation	29.09	[13.63;60.74]	<0.001
Aortic dilatation			
< 39 mm	Ref		
> 39 mm	1.23	[0.89; 1.70]	0.2
Raphe			
None	Ref		
Visible raphe	0.94	[0.70; 1.31]	0.8
Coarctation of the aorta			
Absent	Ref		
Present	0.72	[0.40; 1.32]	0.3

Univariate delayed-entry Cox regression analyses predicting the age-related hazard of surgery of the aortic valve or proximal aorta. Coarctation of the aorta is included regardless of repair status. P values from Wald’s test

*HR* hazard ratio, *CI* confidence interval

## Discussion

The primary finding of this study is that the two-sinus BAV morphology was associated with a 46% higher age-adjusted likelihood of requiring surgery of the aortic valve or ascending aorta than fused BAV. Secondly, the increased hazard among two-sinus BAV patients was present from mid-age and onwards, coinciding with the age that most BAV patients typically begin to develop surgery requiring valve dysfunction or aortic dilatation. When considering the specific BAV phenotype, it seemed that patients with the two-sinus BAV with antero-posterior orientation had a particularly unfavorable prognosis; however, confidence intervals were too broad to conclude on prognostic implications of the specific phenotypes.

Clinical data on the prognostic association of the two-sinus BAV was emphasized as a gap in knowledge by the BAV experts introducing the International Consensus Classification and Nomenclature in 2021. This classification provides a standardized language for clinicians and researchers working with the BAV condition [13]. We observed a prevalence of the two-sinus BAV of 16.8% in our cohort, which was in line with two recently reported outpatient cohorts [19, 20]. That a two-sinus morphology was associated with a worse prognosis differed from our a-priori expectation, as two previous studies have associated the presence of a raphe with surgical complications in BAV disease. Kong et al. used the classification proposed by Sievers and Schmidtke and found that a visible raphe was associated with a higher unadjusted rate of surgery of the aortic valve or aorta [8, 16]. In another study, Bellino et al. used the classification proposed by Schaefer et al. and associated a visible raphe with a higher 3-year risk of surgery [12, 21]. Both studies used time since echocardiogram as time scale. In our study, we use age as the underlying timescale, as it more accurately reflects the beginning of the pathophysiological process in BAV disease. Using this timescale effectively adjusts for age-differences between subgroups. Accounting for delayed entry adjusts for selection bias, as patients who experienced the event before study entry are not included. These methodological differences may explain why the presence of a raphe was not associated with timing of surgery in our study. A raphe is not always visible on early echocardiograms and may be highly correlated with age-related valve degeneration at time of echocardiographic examination [9].

Interestingly, the unfavorable prognosis of the two-sinus BAV subgroup found in the primary analysis was not apparent when assessing the age-adjusted odds of moderate-severe valve dysfunction and aortic dilatation at index echocardiogram (Fig. 5). On the contrary, the fused BAV morphology was found to have a higher prevalence of moderate-severe aortic regurgitation across all age-groups. These findings are in line with previous studies [10, 21]. However, since the observed association between morphology and timing of surgery must be mediated by an effect of morphology on development of valve dysfunction or aortic dilatation, it does seem counterintuitive. However, we must keep in mind that echocardiographic status at index, although adjusted for age-group, does not directly reflect the rate at which dysfunction/dilatation develops over time. Furthermore, the echocardiographic categories are quite broad and may not correctly identify the highest-risk patients. In our view, the key takeaway message is that the association between BAV morphology and timing of surgery is driven by multiple causal factors rather than a single one.

Lastly, patients with two-sinus BAV had a much higher prevalence of concomitant aortic coarctation. Whereas only 12% of patients with fused BAV had concomitant aortic coarctation, the prevalence was 24% in patients with two-sinus BAV and as high as 43% in those with the two-sinus BAV with antero-posterior phenotype. This observation is in line with a previous study by Lim et al, linking concomitant aortic coarctation with the Sievers type 0 BAV type—a morphology with close resemblance to the two-sinus BAV [22]. In the study by Lim et al., BAV patients with concomitant aortic coarctation were much younger at the time of aortic valve surgery, whereas aortic coarctation was not associated with the timing of surgery in our study. This difference could be due to different study populations given that the study by Lim et al. was based on a surgical database, whereas ours is an outpatient cohort. The prognostic impact of concomitant aortic coarctation in BAV disease needs further exploration in future prospective studies.

### Limitations

Although our study population included as many as 1004 patients with BAV, the length of follow-up reduces the statistical power and especially limits our ability to conclude about absence of associations. Using age as the time scale in our primary analyses enabled us to evaluate how BAV morphology is associated with disease progression over time regardless of the limited follow-up time. Although confidence intervals of the analyses are relatively broad, we must keep in mind that the estimated HR of 1.46 corresponds to the average difference between cumulative hazard curves seen in Fig. 3 [23]. Since the curves diverge progressively from age 45 and onwards, the average HR underestimates the difference between patients in the higher age groups but overestimates the difference in young adults. However, since our cohort includes only those who remained surgery-free until adulthood, we may miss risk differences in early adulthood. Therefore, we cannot exclude the possibility that two-sinus morphology is also associated with prognosis in young adults or children. As is the case in most observational studies, our study is subjected to selection bias. A possible source of bias is misclassification of aortic valve anatomy. We took several measures to minimize potential misclassification, such as reviewing all prior examinations, including more advanced imaging (transesophageal echocardiography and cardiac MR) when available, and reaching consensus agreement in difficult cases. However, we still had to exclude 31 patients where anatomical classification was not possible. Furthermore, it is important to note that our study population consists of BAV outpatients, and thus, our findings may not be generalizable to individuals in which the BAV condition remains undiscovered. However, this selection bias affects the majority of studies in BAV disease and, in our opinion, does not hinder clinical inference from our findings. The proportion of patients requiring surgery during follow-up (20.2%) was high, especially since median follow up was 2.2 years, and this partially reflects that two of three participating departments receive patients referred for surgical evaluation in addition to stable BAV patients from within their catchment area. Therefore, absolute risk estimates in this study are highly dependent on the selection of our cohort and cannot necessarily be generalized to other outpatient populations. However, we find it unlikely that these factors could bias the assessment of the relationship between BAV morphology and the timing of surgery, which was the aim of our study. Unfortunately, we were not able to account for censoring due to emigration from Denmark, as emigration status is not available in the data sources used. However, considering the outpatient status of the study population and the length of follow-up, we expect minimal emigration in the study population during the study period.

## Conclusions

The two-sinus BAV morphology was associated with a higher age-related likelihood of requiring surgical and endovascular procedures on the aortic valve or ascending aorta. These observational data suggest that BAV morphology may have a role in risk stratification of patients with bicuspid aortic valve under clinical surveillance. Furthermore, we observed a high prevalence of concomitant aortic coarctation in patients with two-sinus BAV which emphasizes the importance of complete aortic assessment at diagnosis. Furthermore, our findings imply that BAV morphology, as defined in the International Consensus Classification, should be considered in future prognostic studies of BAV disease.

## Supplementary Information

Below is the link to the electronic supplementary material.

Supplementary file1 (DOCX 25.7 KB)

## Data Availability

Currently, sharing of anonymized data from this study requires a formal data sharing agreement approved by the Region Zealand. We encourage interested researchers to contact the corresponding author for collaboration on this matter.
