# Supplementary material for: Valve morphology and timing of surgery in bicuspid aortic valve disease
Source: Clin Res Cardiol. 2025 Aug 19;115(7):1166–77. doi: 10.1007/s00392-025-02725-1 (PMC13249721; doi:10.1007/s00392-025-02725-1)
Supplement: Supplementary file 1 — Supplementary file1 (DOCX 25.7 KB) [file 392_2025_2725_MOESM1_ESM.docx]

**Supplementary Materials**

|  | **Fused R-L** | **Fused R-N** | **Fused L-N** | **2-sinus L-L** | **2-sinus A-P** | ***p*** |
| --- | --- | --- | --- | --- | --- | --- |
|  | 669 | 144 | 22 | 90 | 79 |  |
| **Male sex** | 497 (74.3) | 98 (68.1) | 17 (77.3) | 63 (70.0) | 60 (75.9) | 0.5 |
| **Age** | 53.9  [40.4, 64.0] | 50.8  [37.7, 61.2] | 65.8  [54.9, 73.3] | 51  [42.6, 64.6] | 39.3  [28.4, 53.2] | <0.0001 |
| **Moderate-severe valve dysfunction** |  |  |  |  |  |  |
| *None* | 301 (45.0) | 61 (42.4) | 7 (31.8) | 41 (45.6) | 47 (59.5) |  |
| *Aortic stenosis* | 205 (30.6) | 49 (34.0) | 11 (50.0) | 35 (38.9) | 21 (26.6) |  |
| *Aortis stenosis and regurgitation* | 57 (8.5) | 8 (5.6) | 2 (9.1) | 5 (5.6) | 0 (0.0) |  |
| *Aortic regurgitation* | 82 (12.3) | 20 (13.9) | 0 (0.0) | 3 (3.3) | 8 (10.1) | 0.02 |
| **Aortic dilatation** | 407 (64.7) | 75 (55.6) | 9 (42.9) | 53 (60.2) | 45 (60.8) | 0.1 |
| **Aortic Coarctation** | 92 (13.9) | 3 (2.1) | 4 (18.2) | 6 (6.8) | 34 (43.0) | <0.0001 |

***Supplementary table 1: Clinical characteristics and findings on index echocardiogram according to specific BAV phenotype.*** *Numbers are count (percentages) or median [interquartile range]. P-values are from chi-square test or Kruskal-Wallis test.*

|  | **Total** | **Fused BAV** | **2-sinus BAV** | ***p*** |
| --- | --- | --- | --- | --- |
| Moderate-Severe Aortic stenosis | 401 (41.0) | 339 (41.6) | 62 (38.0) | 0.45 |
| Moderate-Severe Aortic stenosis and LVEF < 40% | 24 (2.4) | 21 (2.5) | 3 (1.8) |  |
| Aortic valve area, cm^2^ |  |  |  |  |
| < 1 | 184 (19.3) | 148 (18.5) | 36 (23.1) |  |
| 1-1.5 | 182 (19.1) | 160 (20.0) | 22 (14.1) |  |
| 1.6-2.5 | 132 (13.8) | 113 (14.1) | 19 (12.2) |  |
| > 2.5 | 361 (37.8) | 300 (37.5) | 61 (39.1) | 0.32 |
| Median AV mean pressure gradient, mmHg | 14 [7.0, 27.5] | 14 [8, 27] | 13 [7.0, 31.2] | 0.5 |
| Median AV max pressure gradient, mmHg | 22 [12, 41] | 22 [12, 41] | 20 [11.0, 39.5] | 0.4 |
| Moderate-Severe aortic regurgitation | 189 (19.1) | 173 (21.0) | 16 (9.6) | 0.003 |

***Supplementary table 2: Additional measures from index echocardiogram according to Major BAV type.*** *Numbers are count (percentages) or median [interquartile range]. P-values are from chi-square test or Wilcoxon-rank-sum test. LVEF: Left ventricular ejection fraction. AV: Aortic valve.*
